# Primary extraskeletal osteosarcoma of omentum majus

**DOI:** 10.1186/1477-7819-9-25

**Published:** 2011-02-19

**Authors:** Shui-Xiang Tao, Guo-Qin Tian, Meng-Hua Ge, Cheng-Long Fan

**Affiliations:** 1 Department of Surgery, Shaoxing County Center Hospital, Shaoxing, Zhejiang Province, PR China; 2 Department of Gynecology, Shaoxing County Center Hospital, Shaoxing, Zhejiang Province, PR China; 3 Department of General Surgery, Shaoxing County Center Hospital, Shaoxing, Zhejiang Province, PR China; 4 Department of Pathology, Shaoxing County Center Hospital, Shaoxing, Zhejiang Province, PR China

## Abstract

Extraskeletal osteosarcoma is a rare malignant soft tissue tumor. Here we present a case of a primary extraskeletal osteosarcoma arising from omentum majus in a 40-year-old Chinese woman. Ultrasonography of the pelvic cavity showed a large soft tissue mass with marked calcification. Complete surgical resection of the primary tumor was performed and the histopathological diagnosis was extraskeletal osteosarcoma of omentum majus. She was followed up without adjuvant radiotherapy and chemotherapy, and died from widespread intra-abdominal, lung and liver metastases 7 months postoperatively.

## Background

Extraskeletal osteosarcoma (ESOS) is a rare malignant mesenchymal neoplasm in soft tissues but not directly attached to the skeletal system. Fewer than 300 cases have been reported to date. Here, we describe a case of a primary ESOS arising from omentum majus. To the best of our knowledge, this is the second reported case in the English literature.

## Case history

A 40-year-old woman was admitted to our hospital complaining of lower abdominal pain with nausea and vomiting for 4 days. She denied any history of other previous abdominal injuries or pain and had no other pertinent past medical history. The patient's general condition was good and no weight loss was observed. Ultrasonography of the pelvic cavity showed a soft tissue mass with marked calcification, measuring 6.9 × 4.6 cm. Laboratory tests revealed an increase in the alkaline phosphatase (213 u/L) and carbohydrate antigen 125 (102 u/moL). For resection of the tumor, en bloc mass excision with segmental omentum majus resection was performed. During the operation, the mass was located in the lower margin of greater omentum, and tumor invasion into surrounding organs was not observed. There was no lymph node swelling or peritoneal dissemination. About 500 ml of bloody effusion was present in the peritoneal cavity. Frozen section showed a malignant mesenchymal tumor of omentum majus.

**Figure 1:**
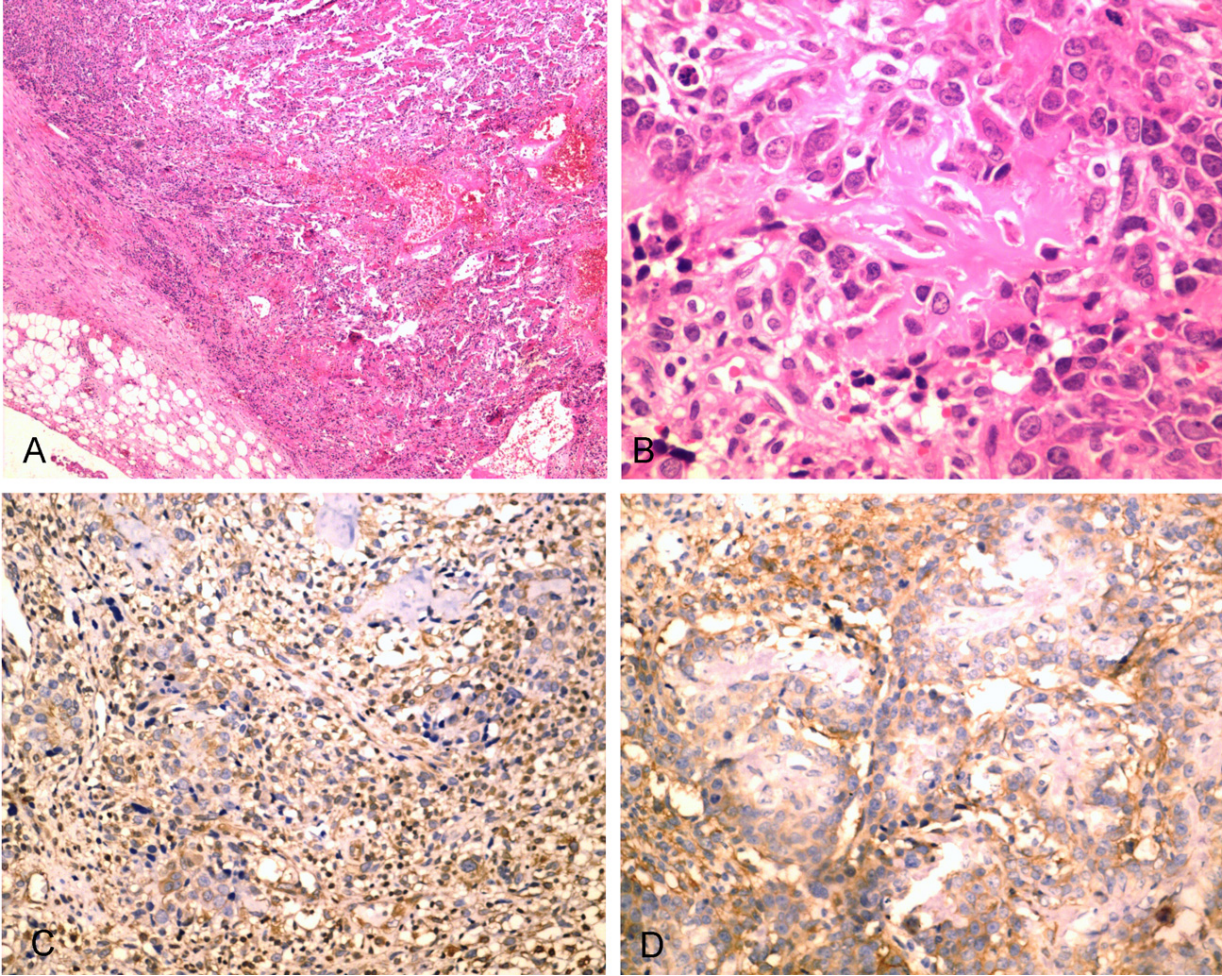
**Histological section (A) showing tumor arising from the omentum (×40, H&E); B) showing calcificied neoplastic osteoid among the tumour cells and osteoclastic giant cells (×400, H&E))**. Immunohistochemical section showed intensive positive reaction with Vimentin (C) and CD99 (D) (×200).

The resected tumor was 6.0 × 4.0 × 3.0 cm in size. The cut surface consisted of a gray-white to tan-yellow solid area with a gritty sensation. Microscopically, there was calcificied neoplastic osteoid among the tumor cells and osteoclastic giant cells that showed intensive positive reaction with Vimentin and CD99 (histiocytic marker) (Figure 1), but negative for CK, CR, EMA, CD117, CD34, CD68, SMA and Desmin. The final pathological diagnosis was extraskeletal osteosarcoma of omentum majus.

**Figure 2:**
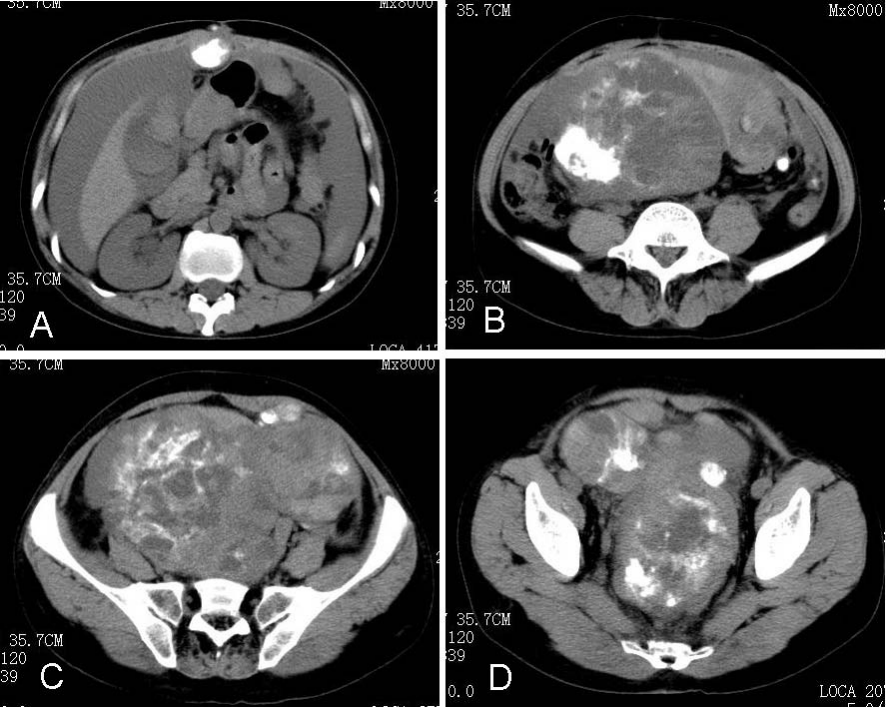
**Abdominal CT revealed a large mass with mottled calcification, and effusions in the peritoneal cavity**.

The postoperative course was uneventful. We recommended adjuvant radiotherapy and chemotherapy, but the patient refused. Five months after the operation, she was readmitted to our hospital due to abdominal pain. Abdominal CT revealed a large mass with mottled calcification and effusions in the peritoneal cavity (Figure 2). She died with widespread intra-abdominal, lung and liver metastases 7 months postoperatively.

## Discussion

Primary tumors develop in the greater omenturn is very rare. Most common histopathologic types are leiomyosarcoma, leiomyoma, gastrointestinal stromal tumor, and teratoma. ESOS is a rare malignant neoplasm arising from soft tissue that produces osteoid, without any continuity to skeletal bones and constitutes 4% of osteosarcomas and 1.2% of all soft-tissue sarcomas [1,2]. ESOS occurs mostly in soft tissue of the thigh, followed by the upper limb and retroperitoneum [3,4]. Cases of ESOS arising in unusual sites, such as the larynx, kidney, esophagus, small intestine, liver, heart, urinary bladder, parotid, and breast have also been reported [2]. In a review of the literature, there is only one report of extraskeletal osteosarcoma arising from the omentum majus [5].

Unlike conventional osteosarcoma of bone, which usually occurs in the first or second decade of life, ESOS occurs predominantly in patients older than 40 years of age [1,3]. The patient reported here was just 40 years old. As with most other tumors, the etiology of ESOS is essentially unknown. It has been reported to have occurred in previously irradiated areas [6], and mechanical injury has also been considered as a causative agent [7]. However, our patient has no history of surgery or regional radiation treatment.

The diagnosis is generally delayed because symptoms are often absent or vague. Typically, the tumor presents as a progressively enlarging soft tissue mass associated with pain in approximately one-third of patients. Serum alkaline phosphatase levels may be elevated. Imaging studies show soft tissue masses with internal cloud-like calcifications or ossification. Histopathologically, there are 2 microscopic components, sarcomatous cells and extracellular matrix, consisting of osteoid or immature bone [8]. Immunohistochemically, the expression of antigens varies in the reported cases. Our case expressed CD99 and vimentin, but was negative for CK, CR, EMA, CD117, CD34, CD68, SMA and Desmin.

The prognosis of ESOS is grave with a cause-specific survival rate at 5 years less than 25% [9]. Radical resection appears to be the best therapeutic option for local control but has no effect on distant metastasis [10]. Hence, systemic chemotherapy is commonly advocated, although the efficacy has not been evaluated with controlled clinical trials due to the rarity of cases. Radiotherapy may provide temporary palliation [11]. Goldstein-Jackson et al. [11] reported that ESOS is better treated with more aggressive multi-agent chemotherapy. In our case, we performed a wide marginal resection, but the patient refused chemotherapy and radiotherapy. More information needs to be obtained concerning the clinical outcome for appropriate management, planning, and prognostic estimation.

## Conclusion

In conclusion, we have reported an exceedingly rare case of extraskeletal osteosarcoma arising from omentum majus. The diagnosis of ESOS should be considered when one encounters a large soft tissue mass that shows abundant intratumoral calcification or ossification. The tumor recurred and spread rapidly despite wide surgical resection, showing highly aggressive biologic behavior and causing death within 7 months.

## Consent

Written informed consent was obtained from the patient for publication of this case report and accompanying images. A copy of the written consent is available for review by the Editor-in-Chief of this journal.

## Competing interests

The authors declare that they have no competing interests.

## Authors' contributions

S-XT prepared the manuscript and reviewed the literature. MHG and G-QT provided the clinical data and reviewed the manuscript. C-LF reviewed the slides and supervised the preparation of the manuscript.
